# Association of urine autoantibodies with disease activity in systemic lupus erythematosus

**DOI:** 10.3389/fmed.2024.1346609

**Published:** 2024-01-19

**Authors:** Yuxian Zhang, Xiaoxia Qu, Lishui Wang, Lijun Song

**Affiliations:** ^1^Department of Rheumatology, Qilu Hospital, Shandong University, Jinan, China; ^2^Qilu Hospital, Shandong Provincial Clinical Research Center for Immune Diseases and Gout, Jinan, China; ^3^Department of Clinical Laboratory, Qilu Hospital, Shandong University, Jinan, China

**Keywords:** systemic lupus erythematosus, lupus nephritis, urine antinuclear antibody, urine anti-dsDNA antibodies, SLEDAI

## Abstract

**Objective:**

The presence of urinary autoantibodies in patients with systemic lupus erythematosus (SLE) has been confirmed by several studies; however, the significance of their presence in urine remains unclear. This study aims to further investigate the association between urine autoantibodies and disease activity as well as organ involvement in SLE.

**Methods:**

This cross-sectional study included 89 SLE patients. Data collected included anti-nuclear antibody (ANA), anti-ENA antibodies, and anti-dsDNA antibody levels in both serum and urine, complement (C) 3, C4 levels in serum, SLE disease activity index-2000 (SLEDAI-2000), renal domains of SLEDAI (RSLEDAI) and non-renal SLEDAI (NRSLEDAI).

**Results:**

The rate of positive urine ANA (uANA) was 33.3% (29/87) among the enrolled patients. Compared to the uANA negative group, the positive group exhibited significantly higher SLEDAI-2000 scores (7.85 ± 5.88 vs. 18.69 ± 6.93, *p* < 0.001), RSLEDAI scores [0 (0, 4.0) vs. 12.0 (8.0, 16.0), *p* < 0.001], and NRSLEDAI [4 (2.0, 8.0) vs. 6.0 (4.0, 9.5), *p* = 0.038]. Patients with positive urine anti-Sm antibody demonstrated significantly elevated SLEDAI-2000 scores compared to those who were negative (25.0 ± 8.80 vs. 10.09 ± 6.63, *p* < 0.001). Similarly, they also had higher RSLEDAI [16.0 (12.0, 16.0) vs. 4.0 (0, 8.0), *p* < 0.001] and NRSLEDAI [9.5 (6.0, 13.5) vs. 4.0 (3.0, 8.0), *p* = 0.012], as well as a greater prevalence of renal involvement compared to their negative counterparts (100% vs. 58.2, *p* = 0.022). There was a positive correlation between uANA titer and both SLEDAI-2000 (*r_s_* = 0.663, *p* < 0.001) and RSLEDAI (*r_s_* = 0.662, *p* < 0.001). The serum anti-dsDNA antibody level did not exhibit a significant correlation with RSLEDAI (*r_s_* = 0.143, *p* = 0.182). Conversely, the urine anti-dsDNA antibody level demonstrated a significant positive correlation with RSLEDAI (*r_s_* = 0.529, *p* < 0.001).

**Conclusion:**

Urine ANA is associated with both global SLEDAI and RSLEDAI scores. Urine anti-Sm antibody is associated with an increased incidence of renal involvement in SLE. The urine anti-dsDNA antibody level, rather than the serum anti-dsDNA antibody level, exhibits a significant association with RSLEDAI in SLE.

## 1 Introduction

Systemic lupus erythematosus (SLE) is a complex autoimmune disease characterized by the presence of various autoantibodies, particularly antinuclear antibody (ANA) (1). SLE manifests with diverse clinical features and simultaneous involvement of multiple organs and systems (2). Within the first year of disease onset, approximately 40% of SLE patients will develop organ damage (3), while around 50% will experience permanent organ damage within 5 years (4). Early diagnosis of SLE is crucial for effective treatment. Therefore, biomarkers, especially immunological biomarkers, have emerged as valuable tools for accurate diagnosis, assessment of disease activity, and achieving optimal disease control.

The discovery of lupus cells has garnered significant attention in diagnosing SLE (5). With the identification and development of ANA detected through indirect immunofluorescence (IIF) on Human epithelial type-2 (HEp-2) cells, it has replaced lupus cells as an important component in classifying individuals with SLE (6–8). In fact, positive ANA at any time has been defined as a required entry criterion according to the latest EULAR/ACR-2019 criteria (9).

However, unlike its role in classifying and diagnosing SLE, serum ANA titer is not recommended as a monitoring indicator for disease activity due to uncertain correlation between serum ANA titer and SLE disease activity levels (10). Furthermore, severe relapse of lupus nephritis may be accompanied by the disappearance of ANA (11), indicating that negative conversion or loss of immunofluorescence ANA could suggest unfavorable prognoses for severe patients (12).

Previous studies have demonstrated the presence of urinary ANA (13–15); however, their significance and utility remain unexplored. Do patients with positive urine ANA and/or anti-ENA antibodies exhibit a distinct phenotype that can be utilized in assessing systemic or kidney disease activity? This study aims to further investigate the association between urine autoantibodies and disease activity as well as organ involvement in SLE.

## 2 Patients and methods

### 2.1 Study population

A cross-sectional study was consecutively conducted on 89 systemic lupus erythematosus (SLE) patients at Qilu Hospital of Shandong University between January 2021 and August 2021, all fulfilling the 2019 American College of Rheumatology classification criteria for SLE (9). Patients with other autoimmune diseases, active infections, or malignancies were excluded from the study. The research was approved by the Medical Ethics Committees of Qilu Hospital of Shandong University (ID: KYLL-202008-021), Jinan, China, adhering to relevant guidelines and regulations including the Declaration of Helsinki. Informed consent was obtained from all participants or their legal guardians. Patients did not participate in any aspect of our research design, conduct, reporting or dissemination plans.

### 2.2 Data collection

Clinical data such as demographic information, concurrent medication use, disease activity scores and laboratory results were collected upon enrollment. Information retrieved from medical records included age, gender, blood urea nitrogen (BUN), serum creatinine (Cr), cystatin C (CysC), complement levels C3, C4, and C1q in serum; ANA titers; anti-extractable nuclear antigen (ENA) antibody levels; anti-dsDNA antibody levels in serum; routine urinalysis results; urinary protein-to-creatinine ratio (uPCR); and urinary albumin-to-creatinine ratio (uACR). The urine protein was categorized into six levels (−, ±, 1+, 2+, 3+, and 4+) based on the findings from the routine urinary test. SLE patients were classified into lupus nephritis (LN) (uPCR > 0.5) and non-LN (uPCR = <0.5) based on the 2012 ACR guidelines for lupus nephritis (16). SLE disease activity was assessed using SLEDAI-2 K (17). The Renal Systemic Lupus Erythematosus Disease Activity Index (RSLEDAI) encompasses the renal domains of SLEDAI, while the Non-Renal SLE Disease Activity Index (NRSLEDAI) is calculated by subtracting RSLEDAI from total SLEDAI.

### 2.3 Assay of autoantibodies in both serum and urine

Clean-catch midstream urine samples were collected, centrifuged at 1,500 RPM for 5 min to eliminate sediment, aliquoted, and stored at −80°C within an hour of collection.

Serum ANA was detected through indirect immunofluorescence using a HEp-2 Complete Kit following the manufacturer’s instructions (Medical & Biological Laboratories Co., Ltd., Japan). The initial dilution for serum samples was set at 1:80. Samples showing fluorescence underwent serial double dilution and retesting until no fluorescence appeared. Results were recorded as the highest dilution of serum with positive findings. Urine ANA detection employed the same reagent and method as serum ANA testing. After conducting a preliminary exploratory study, we selected a urine stock solution with a dilution of 1:1 as the starting concentration for urine ANA detection and subsequently performed stepwise multiple-of-two dilutions until negative results were obtained in indirect immunofluorescence tests.

Anti-ENA antibodies in serum and urine were detected using an immunodot qualitative test following the manufacturer’s instructions (H&J NovoMed Co., Ltd., Beijing, China). The anti-ENA antibody panel included antibodies against Sm, nRNP/Sm, SS-A(Ro), and SS-B(La) in this study. Serum samples were diluted 100-fold, while urine samples underwent a series of dilution tests with levels ranging from 1:1 to 1:40. Ultimately, the urine stock solution (dilution 1:1) was selected for detecting urine anti-ENA antibodies.

Concentrations of anti-dsDNA antibodies were measured using enzyme-linked immunosorbent assay (ELISA) kits as per the manufacturer’s instructions (Shanghai Kexin Biotechnology Co., LTD, Shanghai, China). Serum and urine samples were diluted by factors of 100 and 2, respectively.

### 2.4 Renal histology

In this study, renal histopathology was performed on 15 patients. The types of renal pathology, acute index (AI), and chronic index (CI) were documented.

### 2.5 Statistical analysis

The statistical software SPSS 20.0 for Windows (SPSS Inc., Chicago, Illinois, United States) was utilized to conduct all data analyses. Descriptive statistics are presented as mean ± standard deviation (SD) or median (25th, 75th percentile), unless otherwise specified. To compare variables between the LN group and non-LN group, independent samples *t*-test or Mann–Whitney U test was employed. For comparisons among multiple groups, one-way ANOVA and Kruskal-Wallis one-way ANOVA were performed accordingly. Spearman’s rank correlation coefficient (*r_s_*) was used to assess the relationships between variables. A nominal *p*-value of less than 0.05 was considered statistically significant.

## 3 Results

### 3.1 Demographic and laboratory characteristics in LN and non-LN patients

The study included a total of 89 patients, all of whom had previously tested positive for IIF-ANA. Among them, 52 patients belonged to the non-LN group while 37 patients belonged to the LN group. Table 1 presents the demographic information, laboratory data, and medication details of these participants. Compared to the non-LN group, the LN group exhibited significantly higher levels of BUN (*p* < 0.001), Cr (*p* = 0.005), CysC (*p* < 0.001), urine anti-dsDNA antibody (*p* < 0.001), SLEDAI-2000 score (*p* < 0.001), as well as lower levels of C3 (*p* = 0.002), C4 (*p* = 0.026), and C1q (*p* = 0.002). There was no statistically significant difference in serum ANA titer and anti-dsDNA antibody level between the two groups (*p* = 0.859, *p* = 0.302 respectively). Furthermore, compared to the non-LN group, a higher dosage of glucocorticoid and a greater percentage of immunosuppressant use were observed in the LN group.

**Table 1:** Demographic and laboratory characteristics in LN and non-LN patients.

	Non-LN (*n* = 52)	LN (*n* = 37)	*p* value
Age (years)	38.73 ± 13.84	40.27 ± 13.87	0.607
Male, *n* (%)	3 (5.8%)	7 (18.9%)	0.086
BUN (mmol/L)	5.03 ± 2.10	9.25 ± 6.36	<0.001
Cr (umol/L)	56.5 (47.75, 66.0)	70.0 (54.5, 103.5)	0.005
CysC (mg/L)	1.15 ± 0.36	1.75 ± 0.90	<0.001
IgG (g/L)	17.47 ± 6.21	14.46 ± 8.27	0.054
IgA (g/L)	3.07 ± 1.20	2.93 ± 1.44	0.612
IgM (g/L)	1.11 ± 0.81	0.73 ± 0.36	0.004
C_3_ (g/L)	0.87 (0.65, 1.10)	0.61 (0.38, 0.89)	0.002
C_4_ (g/L)	0.16 (0.08, 0.23)	0.11 (0.00, 0.16)	0.026
C1q (mg/L)	155.23 ± 37.89	130.28 ± 32.69	0.002
24-h proteinuria	0.14 (0.09, 0.37)	2.53 (1.24, 5.69)	<0.001
ANA titer	640 (160, 1,280)	320 (80, 2,560)	0.859
Anti-ds-DNA antibody (IU/ML)	72.32 (33.41, 139.17)	109.55 (23.73, 692.86)	0.302
Urine anti-dsDNA antibody (IU/ML)	15.05 (2.75, 36.52)	156.28 (51.79, 533.43)	<0.001
Urine ANA, *n* (%)	4 (7.69%)	25 (71.43%)	<0.001
SLEDAI-2000	6.83 ± 4.96	18.22 ± 6.61	<0.001
**Medication**			
Glucocorticoid (mg/d, equivalent to prednisone)	40.0 (15.0, 50.0)	50.0 (32.5, 65.0)	0.008
Immunosuppressant (%)	28.85%	64.86%	0.001

WBC, white blood cell; HGB, hemoglobin; PLT, platelet; BUN, blood urea nitrogen; Cr, creatine; CysC, cystatin C; TP, total protein; ALB, albumin; Ig, immunoglobulin; uIg, urine immunoglobulin; C, complement; SLEDAI, SLE disease activity index.

Glucocorticoids refer to prednisone or methylprednisolone; Immunosuppressants refer to cyclophosphamide, mycophenolate, tacrolimus, cyclosporine, methotrexate or leflunomide.

Normally distributed data are expressed by mean ± SD; The non-normal distribution data are expressed by median (25th, 75th percentile).

The comparison of normally distributed data was conducted using an independent samples *t*-test, while the comparison of non-normally distributed data was performed using the Mann–Whitney U test. Additionally, Chi-Square test or Fisher’s exact test was employed for testing rates.

Due to concerns regarding the potential risks associated with renal puncture and other factors, renal histopathology was performed on only 15 patients in this study. Supplementary Table S1 provides detailed information on the renal pathological type, acute index, chronic index, and related autoantibody test results.

### 3.2 The comparisons of ANA and anti-ENA antibodies in serum and urine

In this study, the positive rate of antinuclear antibodies (ANA) in serum and urine of selected patients was 86.5% (77/89) and 33.3% (29/87), respectively, with 27.6% (24/87) of patients showing ANA positivity in both serum and urine. Additionally, the positive rates of anti-extractable nuclear antigen (ENA) antibodies in serum and urine were found to be 70.8% (63/89) and 74.7% (65/87), respectively, while the concurrence rates for anti-ENA antibodies in both serum and urine were observed to be 63.2% (55/87). Supplementary Figure S1 illustrates the consistency between anti-ENA antibodies detected in serum and urine, including specificities such as anti-Sm antibody, anti-nRNP/Sm antibody, anti-SSA antibody, and anti-SSB antibody. Furthermore, it is noteworthy that five patients exhibited positive results for ANA and urinary presence of anti-ENA antibodies despite negative findings on serum ANA testing; similarly, 10 patients displayed urinary detection of anti-ENA antibodies despite negative results on serum testing.

**Figure 1:**
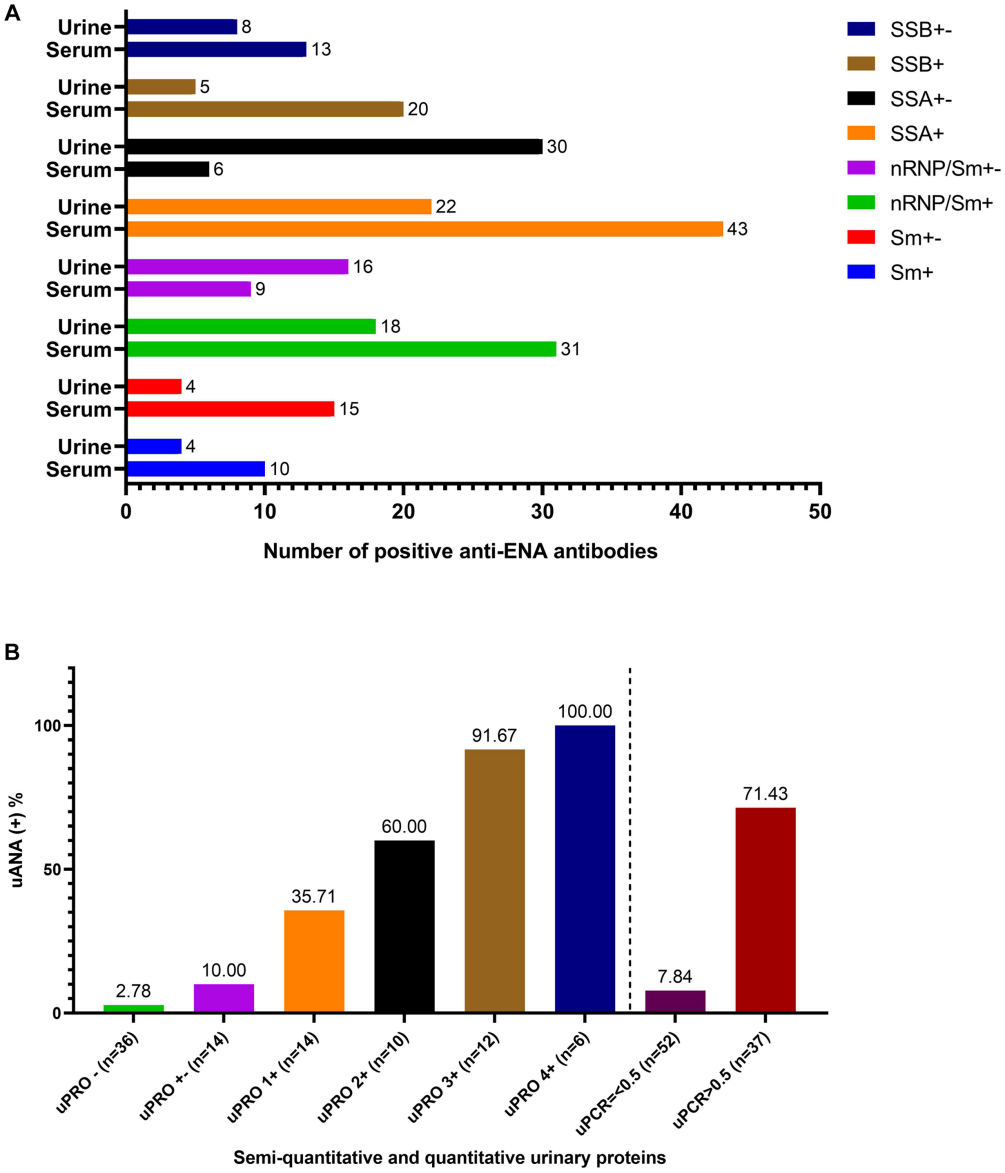
The anti-ENA antibodies in both serum and urine and the ANA in urine. **(A)** The number of the positive anti-ENA antibodies including anti-Sm antibody, anti-nRNP/Sm antibody, anti-SSA antibody, anti-SSB antibody in both serum and urine. **(B)** The positive rate of urinary ANA based on the semi-quantitative determination of urinary protein and uPCR.

As depicted in Figure 1A, the serum exhibited the highest detection rate for anti-SSA antibodies, followed by anti-nRNP/Sm and anti-SSB antibodies, while anti-Sm antibodies displayed the lowest detection rate. Consistently, urine samples demonstrated similar detection rates for these antibodies. Notably, both anti-SSA and anti-nRNP/Sm antibodies were concurrently detected in serum and urine with a higher level of consistency. The correlation between the presence of ANA and the specificities of autoantibodies associated with SLE in serum and urine is presented in Supplementary Table S2.

### 3.3 Urine ANA and anti-ENA antibodies profile in SLE patients with negative ANA and anti-ENA antibodies in serum

Out of the 86 patients who underwent uANA and uENA testing, six patients exhibited negative ANA and anti-ENA antibodies in serum but tested positive for either ANA or anti-ENA antibodies in urine. With the exception of one patient who showed a positive uANA result (1:1), all five remaining patients were positive for urine anti-ENA antibody. Among them, five patients were positive (*n* = 3) or weakly positive (*n* = 2) for anti-SSA antibody, two patients were positive (*n* = 1) or weakly positive (*n* = 1) for anti-nRNP/Sm antibody, and one patient was combined with weakly positive anti-Sm antibody. Furthermore, apart from one patient with negative urinary protein levels, all other five patients demonstrated urinary protein levels greater than or equal to 2+.

### 3.4 The relationship between the detection rate of anti-ENA antibody and different urine dilution

The first 10 patients were selected for the detection of anti-ENA antibodies at urine dilutions of 1:1, 1:10, 1:20, and 1:40, respectively. As presented in Table 2, there was a decrease in the antibody detection rate with increasing urine dilution. Anti-nRNP/Sm antibodies were detected in two patients’ urine samples (diluted at 1:1) despite negative serum anti-nRNP/Sm antibody results; however, when the urine was diluted to 1:10, no anti-nRNP/Sm antibodies were detected. Similar observations were made for anti-SSA antibodies which were found in four urine samples (diluted at 1:1), but disappeared upon further dilution to 1:10. Anti-Sm antibodies were detected in three undiluted urine samples (diluted at 1:1), out of which two became negative when diluted to a ratio of 10-fold while one remained positive even after being diluted by a factor of 40-fold (at a ratio of 40). A strong concordance between the detection results of urinary anti-ENA antibodies at a dilution ratio of 1:1 and those obtained from serum diluted at a ratio of 100-fold was observed.

**Table 2:** Anti-ENA antibodies in both serum and urine from 10 patients with SLE.

Item		Dilution	Pt 1	Pt 2	Pt3	Pt 4	Pt 5	Pt 6	Pt 7	Pt 8	Pt 9	Pt 10
SSA	Serum	1:100	**+**		**+**	**+**	**+**	**−**	**−**	**+**	**+**	**+**
	Urine	1:1	**+**	**+**	**±**	**±**	**±**	**−**	**−**	**±**	**+**	**−**
		1:10	**+**	**+**	**−**	**−**	**−**	**−**	**−**	**−**	**+**	**−**
		1:20	**+**	**±**	**−**	**−**	**−**	**−**	**−**	**−**	**+**	**−**
		1:40	**+**	**±**	**−**	**−**	**−**	**−**	**−**	**−**	**+**	**−**
SSB	Serum	1:100	**+**	**−**	**+**	**−**	**−**	**−**	**−**	**+**	**+**	**+**
	Urine	1:1	**±**	**±**	**−**	**−**	**−**	**−**	**−**	**−**	**−**	**±**
		1:10	**±**	**±**	**−**	**−**	**−**	**−**	**−**	**−**	**−**	**−**
		1:20	**±**	**−**	**−**	**−**	**−**	**−**	**−**	**−**	**−**	**−**
		1:40	**−**	**−**	**−**	**−**	**−**	**−**	**−**	**−**	**−**	**−**
Sm	Serum	1:100	**−**	**+**	**+**	**−**	**−**	**−**	**−**	**−**	**+**	**−**
	Urine	1:1	**−**	**±**	**±**	**−**	**−**	**−**	**−**	**−**	**+**	**−**
		1:10	**−**	**−**	**−**	**−**	**−**	**−**	**−**	**−**	**+**	**−**
		1:20	**−**	**−**	**−**	**−**	**−**	**−**	**−**	**−**	**+**	**−**
		1:40	**−**	**−**	**−**	**−**	**−**	**−**	**−**	**−**	**+**	**−**
nRNP/Sm	Serum	1:100	**−**	**+**	**+**	**+**	**−**	**−**	**−**	**+**	**+**	**−**
	Urine	1:1	**−**	**+**	**±**	**−**	**±**	**−**	**−**	**±**	**+**	**±**
		1:10	**−**	**+**	**−**	**−**	**−**	**−**	**−**	**−**	**+**	**−**
		1:20	**−**	**+**	**−**	**−**	**−**	**−**	**−**	**−**	**+**	**−**
		1:40	**−**	**+**	**−**	**−**	**−**	**−**	**−**	**−**	**+**	**−**

SSA, anti-SSA antibody; SSB, anti-SSB antibody; Sm, anti-Sm antibody; nRNP/Sm, anti-nRNP/Sm antibody; Pt, patient; +, positive; ±, weak positive; −, negative.

### 3.5 The relationship between urine protein and uANA

The positive rate of urinary antinuclear antibodies (uANA) was 71.43% in patients with a uPCR greater than 0.5, as depicted in Figure 1B. The prevalence of uANA positivity increased proportionally with the level of urinary protein excretion. Specifically, the positive rate of uANA was 91.67% in patients with a urinary protein score of 3+ and reached 100% in those with a urinary protein score of 4+. Furthermore, even among patients exhibiting negative or weakly positive urine protein levels (or uPCR<0.5), ANA could still be detected in some cases.

### 3.6 The comparison of laboratory data based on the urine ANA

To investigate the characteristics of patients exhibiting positive urine ANA, we categorized the subjects into two groups based on their urine ANA results: positive and negative. As depicted in Table 3, compared to the uANA-negative group, individuals in the uANA-positive group displayed elevated levels of Cr, BUN, CysC, uPCR, uACR, urine anti-dsDNA antibodies, SLEDAI-2000 scores as well as RSLEDAI and NRSLEDAI scores; additionally, they exhibited decreased levels of C3.

**Table 3:** The comparison of laboratory data based on the urine ANA.

Items	uANA (+) (*n* = 29)	uANA (−) (*n* = 58)	*p* value
BUN (mmol/l)	9.77 ± 6.86	5.38 ± 2.53	0.002
Cr (umol/l)	58.0 (49.25, 66.5)	73.0 (58.5, 127.0)	0.001
CysC (mg/l)	1.85 ± 0.97	1.19 ± 0.38	0.001
IgG (g/l)	15.46 ± 8.64	16.85 ± 6.40	0.400
IgA (g/l)	2.93 ± 1.47	3.05 ± 1.24	0.674
IgM (g/l)	0.74 ± 0.38	1.07 ± 0.78	0.037
uPCR (g/g)	2.65 (1.40, 6.78)	0.1 (0.05, 0.38)	<0.001
uACR (g/g)	1.50 (0.70, 4.66)	0.01 (0.01, 0.09)	<0.001
24-h proteinuria	2.57 (1.55, 6.84)	0.15 (0.10, 0.48)	<0.001
C3 (g/l)	0.54 (0.41, 0.84)	0.87 (0.63, 1.09)	0.001
C4 (g/l)	0.11 (0, 0.16)	0.15 (0.08, 0.24)	0.118
C1q (mg/l)	138.55 ± 35.92	148.65 ± 38.80	0.244
ANA titer	1280.0 (80.0, 2,560)	320 (80, 1,280)	0.185
Anti-dsDNA antibody (IU/ml)	109.55 (23.73, 1117.86)	72.32 (32.56, 150.13)	0.152
Urine anti-dsDNA antibody (IU/ml)	301.41 (68.78, 548.17)	15.49 (3.75, 42.14)	<0.001
SLEDAI-2000	18.69 ± 6.93	7.85 ± 5.88	<0.001
RSLEDAI	12.0 (8.0, 16.0)	0 (0, 4)	<0.001
NRSLEDAI	6.0 (4.0, 9.5)	4.0 (2.0, 8.0)	0.038

BUN, blood urea nitrogen; Cr, creatine; CysC, cystatin C; uPCR, urine protein creatinine ratio; uACR, urine albumin creatinine ratio; C, complement; ANA, antinuclear antibody; uANA, urine antinuclear antibody; SLEDAI, SLE disease activity index; RSLEDAI, renal SLE disease activity index; NRSLEDAI, non-renal SLE disease activity index.

Normally distributed data are expressed by mean ± SD; The non-normal distribution data are expressed by median (25th, 75th percentile).

The comparison of normally distributed data was conducted using an independent samples *t*-test, while the comparison of non-normally distributed data was performed using the Mann–Whitney U test.

### 3.7 The comparison of clinical and laboratory data based on the anti-ENA antibodies

Eighty-seven patients were categorized based on the presence of specific anti-ENA antibodies. As depicted in Table 4, compared to those with negative urinary anti-Sm antibody, the positive group exhibited decreased levels of C3 and C4, elevated BUN, Cr, CysC, serum ANA titers, anti-dsDNA antibody levels, SLEDAI-2000 scores as well as RSLEDAI and NRSLEDAI scores. Additionally, the positive group demonstrated a higher prevalence of serositis and renal involvement when compared to the negative group. Furthermore, analysis based on urine anti-Sm/nRNP antibody results revealed that the positive group exhibited higher serum ANA titers and lower serum creatinine levels, while demonstrating reduced skin involvement compared to the negative group. Patients with positive urinary anti-SSA antibody displayed increased SLEDAI-2000 scores and decreased C3 levels. Urinary anti-SSB antibody was solely associated with heightened serum ANA titer.

**Table 4:** The comparison of laboratory and clinical data based on the uSm, uSm/nRNP, uSSA, and uSSB.

	uSm		uSm/nRNP		uSSA		uSSB	
Items	uSm (+/±) (*n* = 8)	uSm (−) (*n* = 79)	uSm/nRNP (+/±) (*n* = 34)	uSm/nRNP (−) (*n* = 53)	uSSA (+/±) (*n* = 52)	uSSA (−) (*n* = 35)	uSSB (+/±) (*n* = 74)	uSSB (−) (*n* = 13)
BUN (mmol/l)	11.60 (7.3, 15.35)*	5.10 (3.80, 7.30)	4.90 (3.48, 7.35)	5.60 (4.55, 7.75)	5.70 (3.85, 9.33)	4.90 (4.10, 7.30)	5.60 (4.45, 9.0)	5.25 (3.80, 7.50)
Cr (umol/l)	96.5 (61.0, 180.25)*	60.0 (51.0, 77.0)	53.0 (46.0, 67.0)^#^	65.0 (55.0, 88.5)	61.0 (51.0, 87.5)	64.0 (51.0, 77.0)	65.0 (58.0, 99.5)	60.5 (50.75, 78.0)
CysC (mg/l)	2.02 (1.46, 2.70)*	1.20 (1.0, 1.42)	1.14 (0.98, 1.46)	1.27 (1.01, 1.71)	1.23 (1.0, 1.95)	1.20 (1.0, 1.39)	1.27 (1.06, 1.90)	1.21 (1.0, 1.52)
C3 (g/l)	0.43 ± 0.33*	0.81 ± 0.34	0.79 ± 0.40	0.77 ± 0.33	0.71 ± 0.35^&^	0.88 ± 0.34	0.72 ± 0.38	0.79 ± 0.36
C4 (g/l)	0.04 (0, 0.11)*	0.14 (0.08, 0.21)	0.12 (0.08, 0.20)	0.14 (0, 0.23)	0.12 (0.02, 0.19)	0.16 (0.08, 0.26)	0.14 (0.04, 0.21)	0.14 (0.05, 0.21)
Anti-dsDNA antibody (IU/ml)	536.63 (96.33, 1253.29)*	73.29 (28.39, 276.15)	94.78 (23.31, 194.13)	87.33 (32.90, 490.07)	80.06 (26.08, 450.34)	91.59 (35.23, 362.11)	97.60 (48.15, 911.70)	88.52 (25.33, 215.84)
ANA titer	1920 (1,280, 2,560)*	320 (80, 1,280)	1,280 (160, 1,280)^#^	160 (80, 1,280)	640 (80, 2,240)	320 (80, 1,280)	1,280 (640, 2,560)^δ^	300 (80, 1,280)
SLEDAI-2000	25.0 ± 8.80*	10.09 ± 6.63	11.59 ± 10.05	11.38 ± 6.58	12.89 ± 8.77^&^	9.34 ± 6.42	13.15 ± 9.08	11.16 ± 7.90
RSLEDAI	16.0 (12.0, 16.0)*	4.0 (0, 8.0)	2.0 (0, 12.0)	4.0 (0, 10.0)	8.0 (0, 12.0)	4.0 (0, 8.0)	0.0 (0, 14.0)	4.0 (0, 12.0)
NRSLEDAI	9.5 (6.0, 13.5)*	4.0 (3.0, 8.0)	4.0 (3.0, 8.0)	6.0 (3.0, 8.0)	6.0 (3.0, 8.0)	4.0 (3.0, 8.0)	6.0 (3.5, 10.0)	5.0 (3.0, 8.0)
Hematological involvement, *n* (%)	7 (87.5%)	47 (59.5%)	20 (58.8%)	34 (64.2%)	31 (59.6%)	23 (65.7%)	7 (53.8%)	47 (63.5%)
Musculoskeletal involvement, *n* (%)	1 (12.5%)	8 (10.1%)	3 (8.8%)	6 (11.3%)	6 (11.5%)	3 (8.6%)	1 (7.7%)	8 (10.8%)
NPSLE, *n* (%)	2 (25.0%)	5 (6.3%)	3 (8.8%)	4 (7.5%)	6 (11.5%)	1 (2.9%)	1 (7.7%)	6 (8.1%)
Serositis, *n* (%)	6 (75.0%)*	18 (22.8%)	12 (35.3%)	12 (22.6%)	17 (32.7%)	7 (20.0%)	3 (23.1%)	21 (28.4%)
Digestive involvement, *n* (%)	0 (0%)	4 (5.1%)	0 (0%)	4 (7.5%)	3 (5.8%)	1 (2.9%)	0 (0%)	4 (5.4%)
Renal involvement, n (%)	8 (100%)*	46 (58.2%)	17 (50.0%)	37 (69.8%)	32 (61.5%)	22 (62.9%)	8 (61.5%)	46 (62.2%)
Skin involvement, *n* (%)	0 (0%)	22 (27.8%)	3 (8.8%)	19 (35.8%)^&^	11 (21.2%)	11 (31.4%)	6 (46.2%)	16 (21.6%)

uSm, urine anti-Sm antibody; uSm/nRNP, urine anti-Sm/nRNP antibody; uSSA, urine anti-SSA antibody; uSSB, urine anti-SSB antibody; BUN, blood urea nitrogen; Cr, creatine; CysC, cystatin C; C, complement; dsDNA, double-strand DNA; ANA, antinuclear antibody; SLE, systemic lupus erythematosus; SLEDAI, SLE disease activity index; RSLEDAI, renal SLE disease activity index; NRSLEDAI, non-renal SLE disease activity index; NPSLE, neuropsychological SLE.

Hematological involvement refers to leukopenia, autoimmune hemolysis, and thrombocytopenia; Musculoskeletal involvement refers to arthritis and myositis; NPSLE refers to organic brain syndrome, visual disturbance, cranial nerve disorders, lupus headache, and cerebrovascular accidents (CVA); Serositis refers to pleurisy and pericarditis; Digestive system involvement refers to various manifestations associated with SLE such as abdominal pain, diarrhea, intestinal wall edema, and mesenteric vasculitis; Renal involvement refers to hematuria, pyuria, proteinuria, urinary casts, and renal insufficiency; Skin involvement refers to rash, alopecia, and mucosal ulcers.

Normally distributed data are expressed by mean ± SD; The non-normal distribution data are expressed by median (25th, 75th percentile).

The comparison of normally distributed data was conducted using an independent samples *t*-test, while the comparison of non-normally distributed data was performed using the Mann–Whitney U test. Additionally, Chi-Square test or Fisher’s exact test was employed for testing rates.

*Compared to uSm (−), *p* < 0.05; ^#^Compared to uSm/nRNP (−), *p* < 0.05; ^&^Compared to uSSA (−), *p* < 0.05; ^δ^Compared to uSSA (−), *p* < 0.05.

### 3.8 The relationship between serum and urine autoantibody and SLE disease activity

The urine ANA titer exhibited a significant positive correlation with uPCR (*r_s_* = 0.710, *p* < 0.001), uACR (*r_s_* = 0.725, *p* < 0.001), SLEDAI-2000 (*r_s_* = 0.663, *p* < 0.001), RSLEDAI (*r_s_* = 0.658, *p* < 0.001), and NRSLEDAI (*r_s_* = 0.246, *p* = 0.022). Additionally, it demonstrated a negative correlation with C3 (*r_s_* = −0.416, *p* < 0.001) and C4 (*r_s_* = −0.216, *p* = 0.045). Urine anti-dsDNA antibody levels exhibited a significant correlation with C3 (*r_s_* = −0.263, *p* = 0.014), uPCR (*r_s_* = 0.596, *p* < 0.001), and uACR (*r_s_* = 0.610, *p* < 0.001). The serum anti-dsDNA antibody level did not exhibit a significant correlation with RSLEDAI (*r_s_* = 0.143, *p* = 0.182). Conversely, the urine anti-dsDNA antibody level demonstrated a significant positive correlation with RSLEDAI (*r_s_* = 0.529, *p* < 0.001).

## 4 Discussion

Although new biomarkers have made great progress in increasing the accuracy of SLE diagnosis (18) and assessing disease activity (19, 20), conventional biomarkers like anti-dsDNA antibodies (21), and complement factors (22) continue to hold an indispensable role in diagnosing and assessing SLE. In the latest 2019 classification criteria for SLE by EULAR/ACR, anti-dsDNA antibody and complement components remain crucial elements for classification purposes (9).

ANA titers may fluctuate alongside changes in both the underlying disease state of SLE itself as well as immunosuppressant usage. A study on belimumab has indicated that up to 30% of screened SLE patients are found to be ANA negative, implying that clinically significant cases exist especially among those treated with glucocorticoids and immunosuppressants (23). Oh Chan Kwon et al. reported that seroconversion to ANA negativity (titer <1:40) occurred in 9.7% of individuals with SLE. Furthermore, patients who experienced negative seroconversion for antinuclear antibodies exhibited significantly lower risk of systemic lupus erythematosus flare-ups (adjusted hazard ratio 0.13, *p* = 0.007), whereas an increase in ANA titer was associated with elevated risk of experiencing a flare-up in SLE (24). According to general logic, elevation in serum ANA titer often signifies increased activity within one or more plasma cells present within the patient’s body and consequently suggests heightened disease activity within their case of SLE; however, this is not always true (24, 25) due to various factors such as assay variation (26), substrates used during testing procedures (27), formation of immune complexes (28), and protein loss from kidney (11, 12).

Meanwhile, several studies have confirmed the presence of autoantibodies in urine. N L Meryhew et al. reported that urine ANA could be detected by indirect immunofluorescence (IIF) in 32% of SLE patients using HEp-2 cell substrate. Sixty-two percent of SLE patients with urine ANA had proteinuria (13). Williams et al. (14) and Macanovic et al. (15) reported the detection of anti-DNA antibodies in the urine of lupus nephritis patients. Consistent with these findings, our present study observed a positive rate of urinary ANA at 33.3%, which increased gradually with increasing urinary protein levels. Even among SLE patients with urinary protein at 1+, more than one-third showed detectable levels of ANA in their urine, suggesting that urinary ANA is a common phenomenon in LN patients. However, prior investigations have not explored the significance associated with the presence of autoantibodies in urine.

As shown in Table 3, individuals within the uANA-positive group exhibited elevated levels of both urine anti-dsDNA antibodies and disease activity scores including SLEDAI-2000 score as well as RSLEDAI score and NRSLEDAI score compared to the negative group. Moreover (in line with this), there was a significant positive correlation between uANA titers and both SLEDAI scores as well as RSLEDAI scores; indicating that uANA is associated with SLE total disease activity and renal involvement.

Anti-dsDNA antibodies exhibit high specificity for systemic lupus erythematosus (SLE) and are detectable in the bloodstream years before clinical diagnosis (29). Numerous studies have investigated the role of anti-dsDNA antibodies in kidney injury associated with SLE (30–32). Levels of anti-dsDNA antibodies correlate with disease activity, particularly in patients with lupus nephritis (33, 34). The strong association between serum anti-dsDNA antibodies and SLE disease activity has been extensively established (35). Pan et al. discovered that an upsurge in anti-dsDNA titer predicted the subsequent development of a severe SLE flare within 6 months. However, an increase in anti-dsDNA titer did not serve as a predictive factor for renal flares (36). This implies that there is no inherent association between serum levels of anti-dsDNA antibodies and renal involvement in SLE. Since elevated serum levels of anti-dsDNA antibodies are linked with disease activity, it would be reasonable to anticipate increased levels during episodes of lupus nephritis flares. Surprisingly, Ho, A et al. found that serum levels of anti-dsDNA antibodies did not escalate but rather decreased during these flares (37), further supporting the absence of correlation between serum antibody levels and the flares of lupus nephritis. Similar to the findings of the two studies above, there was no significant association between serum anti-dsDNA antibody levels and renal disease activity (RSLEDAI) scores in our current study.

However, our study demonstrated significantly higher levels of urine anti-dsDNA antibodies in the LN group, consistent with previous reports on excretion of anti-dsDNA antibodies in urine (14, 28, 38). In the present study, urine anti-dsDNA antibody levels exhibited a noteworthy positive correlation with RSLEDAI scores, indicating their potential relevance to renal involvement. Further investigations are warranted to elucidate whether urine anti-dsDNA antibody levels can serve as predictive markers for SLE renal flares.

In addition to the presence of urine ANA and urine anti-dsDNA antibodies, the detection of urinary anti-ENA series antibodies is also highly prevalent. Anti-Sm antibodies exhibit high specificity for SLE and are often detectable prior to diagnosis (29). Immune deposits containing anti-Sm antibodies have been identified in the glomeruli of SLE patients (39). However, the clinical significance of anti-Sm antibodies remains uncertain. One study suggested a correlation between serum anti-Sm antibodies and increased renal involvement (40), while another study found no association between serum anti-Sm antibodies and renal involvement (41). Given these conflicting findings, current evidence does not support the use of anti-Sm antibodies as reliable prognostic or activity markers in SLE. Therefore, it is worth investigating whether urine anti-Sm antibodies are associated with renal involvement. The presence of anti-Sm antibodies in urine primarily results from renal leakage of plasma anti-Sm antibodies. In this study, patients with positive urinary anti-Sm antibody tests exhibited 100% renal involvement, which was significantly higher compared to those with negative urinary anti-Sm antibody tests and those with positive urinary other anti-ENA antibodies, including anti-nRNP/Sm, anti-SSA, and anti-SSB antibodies. This suggests that in patients without renal involvement at diagnosis, a positive urine screening for anti-Sm antibodies may indicate future renal involvement; however, further research is needed to establish its true predictive value.

Meanwhile, according to Table 2, the positive rate of urine anti-ENA antibodies decreases gradually with increasing urine dilution. The gradual decrease or disappearance of urine anti-ENA antibodies after dilution is attributed to the reduction in antibody levels and limitations in reagent detection sensitivity. This suggests that higher dilutions may enhance the specificity of specific antibodies for disease diagnosis and assessment but at the expense of sensitivity.

In clinical practice, some patients with SLE have been treated with glucocorticoids and immunosuppressants for an extended period; however, their serum ANA titers have consistently remained high, while the levels of anti-dsDNA antibody and complement have failed to normalize despite stable disease manifestations. Moreover, seropositivity for ANA has long been a classification criterion for SLE without establishing a correlation between ANA titers and disease activity. Although serum levels of anti-dsDNA antibody were associated with SLE disease activity, no association was found between current serum levels and renal involvement. These findings suggest that neither serum ANA nor anti-dsDNA antibodies or complement accurately reflect the disease activity in SLE. This study highlights the close correlation between urinary ANA and overall SLE disease activity, as well as the association of renal disease activity with both urinary ANA and urinary anti-dsDNA antibody levels. Therefore, it is worth investigating whether urine-based biomarkers such as urinary ANA and anti-dsDNA antibodies can be utilized to assess renal disease activity in SLE patients. Additionally, exploring whether the presence of autoantibodies in urine can predict future disease flares holds significant research potential.

The current study was a single-center, small-sample study, necessitating further expansion of the sample size and inclusion of additional study centers. Additionally, the impact of glucocorticoids and immunosuppressants on antinuclear antibodies has been demonstrated in both laboratory and clinical settings (24). In this study, patients in the LN group received higher doses of glucocorticoids and had a greater proportion of immunosuppressant usage compared to those in the non-LN group, which may have influenced the findings. We made every effort to exclude other diseases as potential causes of proteinuria based on a definitive diagnosis of SLE, following standard clinical practice. As reported by Pisetsky et al. (26), the frequency of ANA negativity varies depending on the assay kit used, highlighting the need for further investigations to determine if our results align with those obtained using different assay kits.

## 5 Conclusion

In conclusion, urine ANA is associated with both global SLEDAI and SLEDAI scores. Urine anti-Sm antibody is associated with increased renal involvement in SLE. Notably, the urine anti-dsDNA antibody level, rather than the serum anti-dsDNA antibody level, exhibits a significant association with RSLEDAI in SLE.

## Data availability statement

The raw data supporting the conclusions of this article will be made available by the authors, without undue reservation.

## Ethics statement

The studies involving humans were approved by Medical Ethics Committees of Qilu Hospital of Shandong University. The studies were conducted in accordance with the local legislation and institutional requirements. The participants provided their written informed consent to participate in this study.

## Author contributions

YZ: Data curation, Formal analysis, Investigation, Methodology, Writing – original draft. XQ: Data curation, Methodology, Writing – review & editing. LW: Data curation, Methodology, Writing – review & editing. LS: Conceptualization, Data curation, Formal analysis, Funding acquisition, Investigation, Methodology, Project administration, Software, Supervision, Validation, Writing – review & editing.

## Supplementary material

Supplementary Figure S1 Comparison of anti-ENA antibodies including anti-Sm antibody, anti-nRNP/Sm antibody, anti-SSA antibody, and anti-SSB antibody in both serum and urine.
