# Supplementary figures and images for: Association of urine autoantibodies with disease activity in systemic lupus erythematosus
Source: Front Med (Lausanne). 2024 Jan 19;11:1346609. doi: 10.3389/fmed.2024.1346609 (PMC10835792; doi:10.3389/fmed.2024.1346609)

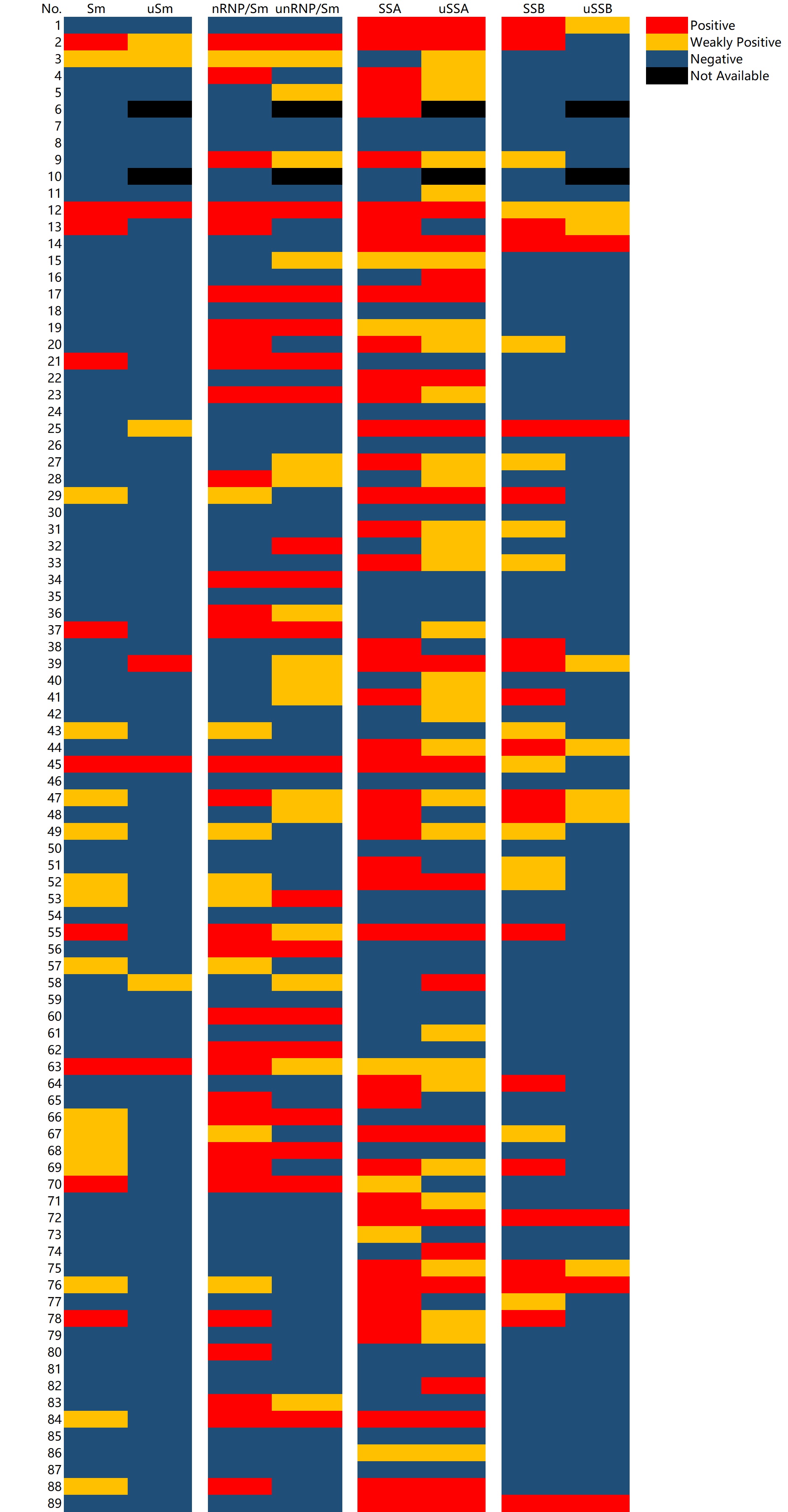

Supplement: Supplementary Figure S1 — Comparison of anti-ENA antibodies including anti-Sm antibody, anti-nRNP/Sm antibody, anti-SSA antibody, and anti-SSB antibody in both serum and urine. [file Image_1.JPEG]
