# Supplementary material for: Association of urine autoantibodies with disease activity in systemic lupus erythematosus
Source: Front Med (Lausanne). 2024 Jan 19;11:1346609. doi: 10.3389/fmed.2024.1346609 (PMC10835792; doi:10.3389/fmed.2024.1346609)
Supplement: Supplementary file 2 [file Table_1.DOCX]

| No. | Serum | | | Urine | | | Renal pathological classification | AI | CI |
| --- | --- | --- | --- | --- | --- | --- | --- | --- | --- |
|  | ANA | Anti-ENA antibodies* | Anti-dsDNA antibody level | ANA | Anti-ENA antibodies | Anti-dsDNA antibody level |  |  |  |
| 1 | Negative | Negative | 549.77 | Negative | Negative | 15.02 | IV | 7 | 7 |
| 2 | 1:160 | Negative | 86.83 | 1:1 | SSA | 301.41 | III+V | 4 | 3 |
| 3 | 1:80 | Negative | 16.21 | 1:1 | SSA (+-), nRNP/Sm (+-) | 283.64 | IV+V | 5 | 8 |
| 4 | Negative | Negative | 21.76 | 1:4 | SSA (+-) | 614.39 | V | 3 | 8 |
| 5 | 1:2560 | nRNP/Sm (+-), Sm (+-), SSB (+-) | 1815.58 | 1:1 | Negative | 291.54 | III+V | 6 | 7 |
| 6 | 1:2560 | SSA (+), SSB (+-), nRNP/Sm (+), Sm (+) | 95.9 | 1:32 | SSA (+), nRNP/Sm (+), Sm (+) | 544.14 | IV | 7 | 2 |
| 7 | 1:80 | Negative | 91.59 | Negative | Negative | 7.0 | III+V | 10 | 5 |
| 8 | 1:2560 | SSA (+), SSB (+), nRNP/Sm (+), Sm (+) | 1673.2 | 1:8 | SSA (+), nRNP/Sm (+-) | 847.39 | IV | 9 | 4 |
| 9 | Negative | Negative | 17.32 | 1:4 | SSA (+), nRNP/Sm (+-), Sm (+-) | 555.15 | III+V | 6 | 6 |
| 10 | 1:1280 | SSA (+-), nRNP/Sm (+), Sm (+) | 652.77 | 1:2 | nRNP/Sm (+) | 66.02 | IV+V | 10 | 2 |
| 11 | Negative | Negative | 13.55 | Negative | Negative | 15.97 | V | 2 | 3 |
| 12 | 1:1280 | SSA (+), nRNP/Sm (+), Sm（+-） | 18.62 | Negative | SSA (+), nRNP/Sm (+) | 63.72 | III+V | 6 | 3 |
| 13 | 1:80 | Negative | 51.95 | Negative | Negative | 151.34 | III+V | 6 | 3 |
| 14 | 1:640 | SSA (+-) | 32.99 | 1:1 | SSA (+-) | 174.05 | III+V | 2 | 1 |
| 15 | 1:2560 | Negative | 555.68 | 1:4 | Negative | 365.59 | IV+V | 10 | 4 |

**Supplementary Table 1** Autoantibodies profile in both serum and urine and renal pathological classification of the lupus nephritis (n=15)

ANA：antinuclear antibody; SSA: anti-SSA antibody; SSB: anti-SSB antibody; nRNP/Sm: anti-nRNP/Sm antibody; Sm: anti-Sm antibody

*Anti-ENA antibodies including anti-SSA antibody, anti-SSB antibody, anti-nRNP/Sm antibody and anti-Sm antibody

AI: acute index; CI: chronic index
