# Supplementary material for: Association of urine autoantibodies with disease activity in systemic lupus erythematosus
Source: Front Med (Lausanne). 2024 Jan 19;11:1346609. doi: 10.3389/fmed.2024.1346609 (PMC10835792; doi:10.3389/fmed.2024.1346609)
Supplement: Supplementary file 3 [file Table_2.DOCX]

|  | Spearman correlation | P value | Kappa | P value |
| --- | --- | --- | --- | --- |
| SSA-uSSA | 0.533 | <0.001 | 0.531 | <0.001 |
| SSB-uSSB | 0.536 | <0.001 | 0.447 | <0.001 |
| nRNP/Sm-unRNP/Sm | 0.443 | <0.001 | 0.438 | <0.001 |
| Sm-uSm | 0.237 | 0.027 | 0.190 | 0.027 |
| ANA-uANA | -1.160 | 0.138 | -0.073 | 0.135 |

Supplementary Table 2 The correlation between the presence of antinuclear antibodies (ANA) and specificities of autoantibodies associated with systemic lupus erythematosus (SLE) in serum and urine

SSA: anti-SSA antibody; SSB: anti-SSB antibody; nRNP/Sm: anti-nRNP/Sm antibody; Sm: anti-Sm antibody; ANA: antinuclear antibody; uSSA: urine anti-SSA antibody; uSSB: urine anti-SSB antibody; unRNP/Sm: urine anti-nRNP/Sm antibody; uSm: urine anti-Sm antibody; uANA: urine antinuclear antibody

The Spearman's rank correlation coefficient was employed for conducting the non-parametric test to analyze the correlation, while the Kappa test was utilized to assess the consistency between serum and urine test results.
